# Psychiatric Comorbidity in Childhood and Adolescence Headache

**DOI:** 10.1007/s11916-015-0479-y

**Published:** 2015-03-10

**Authors:** Grete Dyb, Synne Stensland, John-Anker Zwart

**Affiliations:** 1 Norwegian Centre for Violence and Traumatic Stress Studies, P.B. 181, Nydalen 0409, Oslo, Norway; 2 Institute of Clinical Medicine, Faculty of Medicine, University of Oslo, Oslo, Norway; 3 Department of Neurology and FORMI, Oslo University Hospital, Oslo, Norway

**Keywords:** Headache, Migraine, Tension-type headache, Psychological distress, Depression, Anxiety, Comorbidity

## Abstract

Primary headaches among children and adolescents have a substantial impact on quality of life, daily activities, social interaction, and school performance in combination with psychopathological symptoms. The main purpose of the present paper is to summarize clinical and epidemiological evidence for psychiatric comorbidity among children and adolescents with headaches, to describe how evidence in headache research suggest different pathways involved in the development and maintenance of these comorbid conditions, and finally suggest some elements professionals may find helpful to assess the scope of complaints, related functional impairment, and potential precipitating factors in planning of more targeted treatments.

## Introduction

Childhood is a particularly vulnerable stage in life in terms of onset of health problems, due to the interacting physical, psychological, and social developmental challenges. Recurrent headaches are among the most common somatic complaints and recognized as a significant health problem in this age group [1, 73]. It is well known that headache commonly co-occur with other somatic conditions like musculoskeletal pain, obesity, epilepsy, asthma and allergies [54, 65, 85], as well as psychopathological symptoms [11, 12, 14, 44, 63, 69, 99]. Research is less conclusive on which pathways that may be involved in the development of these comorbid conditions in young headache sufferers [52, 65]. The high levels of impairment in children and adolescents suffering from headache and psychological problems raise the question of how these children should be met by professionals. Previous reports draw attention to the discrepancy between extensive use of clinical resources [42], and rather low success rate for this group of children [52], which calls for new innovative interventions targeting both somatic and psychological/behavioral complaints [6, 27, 56, 81].

The objectives of this paper are as follows:

1. To summarize clinical and epidemiological evidence for psychiatric comorbidity in headaches of children and adolescents.
2. To describe how evidence in headache research suggests different pathways involved in the development and maintenance of these comorbid conditions in young headache sufferers.
3. Suggest some elements professionals may find helpful to assess the scope of complaints, related functional impairment and potential precipitating factors in planning of more targeted treatments.

## Evidence for Psychiatric Comorbidity

### Gender and Developmental Factors

The higher overall prevalence and severity of headaches, specifically migraine and frequent headache, among girls may in part be explained by developmental factors. During early childhood, recurrent headache distributes fairly evenly across gender [41, 51]. In this period, the physiology, psychology, and sociocultural role expectations tend to be more similar for girls and boys than after the onset of puberty. Young children mainly live within the boundaries of the family, day care center and school, and are fully dependent on the social and physical resources they provide. Adolescents have a broader access to the social and physical world beyond close relationships, and the differences in hormone-profile and physiology between females and males accelerate in puberty. In addition, gender-based differences in exposure to potential risk factors in adolescence, such as sociocultural role expectations, limitations, lifestyle, and psychosocial development [21, 89, 100, 104] may increase girls’ susceptibility to recurrent headache. Altogether, these interacting factors related to the onset of adolescence may influence on girls’ susceptibility to internalizing symptomatology, both persistent pain and psychological symptoms [9].

### Methodological Challenges in Measuring Psychiatric Comorbidity

One of the challenges in comparing research on psychiatric comorbidity in children and adolescents is the diversity of applied measurements. Some clinical studies have used categorical assessments of psychiatric comorbidity defined by diagnostic criteria (Diagnostic and Statistical Manual of Mental Disorders (DSM) [10] or The International Classification of Diseases (ICD) [116]), while the majority of studies have applied dimensional measures of psychological and behavioral problems in childhood [62]. The use of categorical assessments of psychiatric comorbidity defined by diagnostic criteria may seem like the most valid choice of method. However, it is well recognized that psychiatric diagnoses in childhood may fail to capture clinical levels of psychopathology, and dimensional measures have been recommended as it may improve accuracy [62, 88]. Including measures for psychiatric diagnostics in headache studies of children may also represent a methodological challenge. Psychiatric diagnostics for children often require information from multiple sources (the child/adolescent, parents, and teachers) and expertise on childhood psychopathology. Reported dimensional measurements typically include a wide range of emotional and behavioral problems in childhood on a continuous scale, measuring frequency or intensity of symptoms, and indicating clinical levels for symptom scores. The Child Behavior Check List (CBCL) [2], measuring internalizing symptoms (depressive, anxiety, and somatic problems) and externalizing symptoms (attention deficit/hyperactivity, oppositional, and conduct problems) seem to be the most widely used instrument. Other examples are The Strength and Difficulties Questionnaire (SDQ) [72] and Hopkins Symptom Check List [23]. Epidemiological studies typically include shortened scales due to limited space. Most instruments have developed parents and teacher versions in addition to self-report measures, although these are infrequently in use in research, most probably due to lack of resources and complication of design.

### Clinical and Epidemiological Studies on Comorbidity

Clinical and population-based studies show that headache is associated with psychopathological symptoms, including both internalizing and externalizing problems, but whether these symptoms are specifically related to migraine or TTH is not evident [5, 34, 62, 69, 71, 79, 90].

In a recent meta analyses, ten studies using the Child Behavior Checklist were selected, in order to asses internalizing (mainly anxiety and depression) and externalizing (mainly behavioral problems) symptoms in different types of headache versus healthy controls. Higher levels of internalizing symptoms were found in patients with either migraine or TTH, while externalization symptoms were more pronounced among those with migraine when compared to healthy controls. There were, however, no significant differences in psychopathological symptoms between the headache groups [11]. This main finding is in compliance with results from a recent large population-based study among adolescents aged 12–17 years, where internalizing symptoms were associated with both migraine and TTH when compared to headache free [14]. It has been suggested that higher levels of internalizing symptoms observed in clinical studies are driven by other somatic complaints included in the CBCL subscale of internalizing behavior score such as headache-related nausea, dizziness, or tiredness. Thus, internalizing symptoms may represent a consequence of having headache rather than a sign of psychological dysfunctioning [19]. Similar to children with other chronic pain conditions [57], children with frequent and chronic daily headache have higher prevalence rates of psychopathological symptoms [14, 35, 76, 113–115], indicating that headache frequency and severity, rather than type, increase the risk of comorbidity.

## Possible Pathways in Development and Maintenance of Psychopathology

**Fig. 1:**
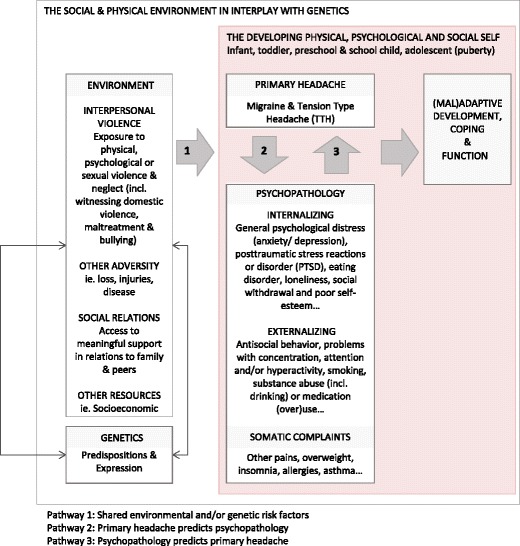
Model of the potential dynamic mechanisms linking primary headache to psychopathology in children and adolescents

Shared risk factors may represent one pathway across somatic and psychological domains as indicated by pathway 1 (Fig. 1). The risk factors for primary headaches may in part share pathophysiological mechanisms [15, 47], reflected in a continuum of clinical severity, ranging from tension-type complaints, through migraine [67], to combined migraine with tension-type headache [111].

Adversities in childhood, such as loss, injuries [53, 93], or lack of social and economic resources within families, in schools and societies [30, 32, 38, 43, 55, 83], seem to be related to increased risk of headache complaints. Recently, exposure to interpersonal violence has been posed as potential triggers of headache in adults [3, 103] and adolescents [31, 33, 97] although documentation is scarce [65, 75].

Access to meaningful support from family and peers enhance belonging and self-esteem [102], while loneliness makes things worse [86]. Loneliness, or perceived social isolation, may result from traumatic exposure [91, 98] and has repeatedly been associated with an increased risk of adverse somatic health outcomes [20, 24, 39, 40, 61, 107]. In children and adolescents, loneliness has been associated with headache [59] and identified as a common trigger of pain [86, 98].

Social support, on the other hand, is a widely recognized protective factor against adverse mental and somatic health outcomes. Early family-based experiences of oneself as an individual who belongs, is loved, protected, and meaningfully supported in the face of need seem to lay ground for our perception of cohesion [84]. Whereas high levels of family cohesion foster resilience in children and adolescents, low levels have been linked to loneliness [109], posttraumatic distress [106], and headache [55]. Thus, family relations may influence on children and adolescents’ ability of coping with pain.

Hence, empirical data suggest that adverse childhood events and psychosocial factors in interplay with genetic predispositions may lead to hyperalgesia and development and maintenance of headaches (Fig. 1) [15, 47, 61, 94]. One suggested mechanism is that pathogenic external stimulation, such as interpersonal violence, triggers threat-related physiological mechanisms including the human stress response system and inducing somatic, psychological, and behavioral reactions. Excessive external threats or internal reactions may over time overwhelm individual capacity, dysregulate related physiological mechanisms, and thereby influence on somatic, psychological and behavioral reactions, and social functioning [22].

The most consistently documented risk factor for psychopathology in childhood is exposure to interpersonal violence [50, 64, 87]. In fact, childhood maltreatment or severe early adversity seem to account for 30 to 70 % of the population attributable risk of all the main categories psychopathology frequently co-occurring with headache, including major depression, anxiety disorders, posttraumatic stress disorder, and substance abuse [64, 101]. Such exposure threatens or violates the child’s or adolescent’s basic needs of physical integrity, sense of safety, belonging and trust in others, and confidence in self-worth and personal capabilities. Common reactions include psychological distress, such as anxiety and depression, as well as social detachment and withdrawal [91].

In addition to environmental factors, shared genetic risk factors may also have an impact on the susceptibility for both headache and psychopathological symptoms. With respect to headache, heritability studies have mostly focused on migraine and a meta-analysis of twin studies estimated the heritability of migraine to 45 % [74], indicating that genetic factors play a substantial role in this familial transmission. In recent years, new genetic risk variants have been identified for psychiatric disorders [48] and for common forms of migraine [7], but so far, little is known about the possible shared genetic risk factors for psychopathological comorbidity in headache though studies indicate that depression and migraine may partly share underlying genetic risk factors [92, 96].

In order to understand how headache may induce psychopathology in children (pathway 2, Fig. 1), it may be useful to dwell over the possible implications of headache in children and young peoples’ lives. Children’s experiences of pain may be particularly threatening, especially in the case of intense, frequent, and non-predictable pain attacks. Such pain may be experienced within a context where the child is unprotected, or where caregivers (and healthcare workers) experience shortcomings and hopelessness. As the head is closely linked to the identity, unexplained headache may be experienced as particularly frightening for the developing child. This “internal threat” may, if not accommodated in terms of safeguarding, induce psychological distress and behavioral reactions [95]. School-aged children and adolescents are particularly vulnerable to frequent and intense headaches impacting concentration, school attendance, and participation in sport and other leisure activities [12, 46]. For some sufferers, the result may be social isolation, feelings of hopelessness, and other depressive symptoms [25]. Altered school results may also impact career plans [110] and ultimately self-esteem and personality traits. Frequency of TTH and migraine was associated with increasing symptoms scores for depression and anxiety in adolescents [14] indicating that headache-free intervals are crucial for mental health functioning.

There is evidence that suggests that psychological distress and behavioral problems can predict the onset or exacerbation of headache (pathway 3, Fig. 1) [78], as well as factors like perceived stress [18] and anger, agitation, or loneliness [86]. Major depression in adolescents, without current or past headache, prospectively predicts onset of headaches in young adulthood [78], while anxiety precedes migraine [66, 112•]. Depressive symptoms are associated with persistence of chronic headache, and psychological and socioeconomic factors predicts new onset CDH [60].

Taken together, psychological distress and headache often co-exist, the conditions share common risk factors, and empirical data suggest that headache may induce psychopathology and vice versa (Fig. 1). The fact that most studies conducted are based on cross-sectional designs and cannot predict causality has drawn the attention to longitudinal studies [11]. Longitudinal studies have provided more solid evidence for bidirectional associations between psychiatric disorders and headache in adults [4, 16, 17].

In children with frequent headache, there is an increased risk of developing psychiatric morbidity in adulthood [30], but there is, however, uncertainty in the literature regarding the direction of the relationship between headache and psychiatric symptoms among adolescents [68, 81]. One longitudinal study showed that preceding anxiety disorders was related to migraine but not TTH [112•]. Another population-based longitudinal study of major depression and migraine showed that respondents with major depressive episodes were 40 % more likely to develop migraine. The association, however, was no longer significant after adjustment for stress and childhood trauma (parental divorce, a lengthy hospital stay, prolonged parental unemployment, frequent parental alcohol, or drug use) [70•]. In a four-year follow-up study of adolescents, higher scores of anxiety and depressive symptoms at baseline were significantly associated with more frequent headache at follow-up, most pronounced for migraine. Among adolescents without recurrent headache at baseline, higher scores for symptoms of anxiety and depression were associated with new onset migraine 4 years later [13•].

## Functional Impairment, Potential Precipitating Factors, and Treatment Strategies

Primary headaches among children and adolescents have a substantial impact on quality of life, daily activities, social interaction, and school performance [37, 49, 58]. Quality of life among headache sufferers may be aggravated by psychopathological symptoms [77], in particular among those with frequent and chronic headache [14, 29, 108, 115].

Despite the uncertainties about the cause and direction of psychopathological symptoms in primary headache disorders, the management by the clinicians confronted with young headache patients should take into account that comorbidity among children and adolescents with headache poses an extra load on their quality of life [77].

In cases of early onset, severe, or refractory headache and co-occurring psychological problems, the history may be key to identify potential factors contributing to onset or maintenance of complaints. In meeting with patients and their families, a thorough assessment of the headache burden (type, frequency, severity, and persistence of symptoms), and function within the family, at school and in relation to peers, forms the basis for a wider dialog around potential hereditary and environmental risk and protective factors. Coping strategies such as use of analgesics, structuring of daily routines, or sleep, should also be assessed. As psychosocial factors seem to influence on trajectories of headache, a thorough social history may be required. Through inclusion of some open-ended questions on lifestyle, family life (structure and cohesion), school environment, and relations to peers, the clinician shows respect and interest for the child or adolescent, builds trust, and gains knowledge of the contextual setting in which the child lives his or her life. Knowledge of parental pain and coping may add to the clinician’s understanding of possible genetic or psychosocial heredity. Specifically, parental factors may impact on children and adolescents coping strategies [26, 36]. As recent studies indicate that psychosocial adversity may contribute to the onset or maintenance of both persistent headache and psychological distress, exposure to violence, sexual abuse, bullying, or neglect may need to be addressed specifically [98]. This information is important in tailoring of individually or family-oriented interventions.

In many cases, it is sufficient applying a simple cognitive approach with emphasis on identifying triggers for headache, modifiable lifestyle factors, and proper advice of medication and acute treatment. In addition, supplementary self-guided cognitive-behavioral self-management strategies might be a promising approach [82, 105]. In patients with frequent and chronic headache, however, one should consider prophylactic medication [45], but the presence of psychopathological symptoms might impact the outcome [28, 35] and accordingly require a combined approach with psychological interventions [8, 27, 80, 81].

Taken together, the phenotypic expression (headache severity and co-occurrence of other somatic and psychological complaints), impact on function and coping, and identified potential risk and protective genetic and environmental factors form the basis for clinical assessment and tailored intervention. Headache is largely a multifactorial disorder, and interventions need to be designed thereafter.
